# Fertility-enhancing effect of oil-based contrast agents during hysterosalpingography and the variation of this effect within a 3-year follow-up period in infertile patients

**DOI:** 10.3389/fmed.2022.948945

**Published:** 2022-08-29

**Authors:** Jingyuan Lu, Dan Qi, Wenjian Xu

**Affiliations:** ^1^Department of Radiological Intervention, Nanjing Maternity and Child Health Care Hospital, Women’s Hospital of Nanjing Medical University, Nanjing, China; ^2^Department of Traditional Chinese Medicine, Nanjing Maternity and Child Health Care Hospital, Women’s Hospital of Nanjing Medical University, Nanjing, China

**Keywords:** hysterosalpingography, oil-based contrast agents, water-based contrast agents, spontaneous pregnancy rate, infertile patients

## Abstract

**Objective:**

The previous study has indicated the fertility-enhancing effect of oil-based contrast agents during hysterosalpingography (HSG) in infertile patients. However, the variation of this effect with the time frame is seldom reported. The current study aimed to explore fertility improvement using oil-based contrast agents and the change of this improvement during the 3-year follow-up period in infertile patients.

**Materials and methods:**

Infertile women who underwent HSG with oil-based contrast agents (*N* = 500) or water-based contrast agents (*N* = 500) were enrolled. Spontaneous pregnancy rate and time to pregnancy were assessed at months (M)1, M2, M3, M6, M12, M24, and M36 after HSG.

**Results:**

The spontaneous pregnancy rate was 79% in the oil-based group and 70.2% in the water-based group. The cumulative spontaneous pregnancy rate was increased in the oil-based group when compared with the water-based group (*p* = 0.015). Fertility-enhancing effect of HSG was increased in the oil-based group when compared with the water-based group at all time points {M1 [odds ratio (OR)]: 1.536}; M2 (OR: 1.455); M3 (OR: 1.494); M6 (OR: 1.356); M9 (OR: 1.288); M12 (OR: 1.249); M24 (OR: 1.131); and M36 (OR: 1.125). While this superiority of the fertility-enhancing effect of HSG in the oil-based group (vs. the water-based group) was decreased with the time frame. Similar findings were also observed based on the physiological cycles.

**Conclusion:**

The HSG procedure with oil-based contrast agents shows a fertility-enhancing effect when compared to water-based contrast agents. This improvement could last at least 1 year while dropping to the normal level within the subsequent 2 years.

## Introduction

Infertility is the inability to conceive after 1 year of unprotected intercourse (1–3). The incidence of infertility has been continuously increasing in recent decades, and its prevalence is as high as 13.00–24.58% in women with fertility intentions (3, 4). Infertility women suffer from a vast disease burden, including physical distress and emotional devastation, which might cause physiological disorders for infertile women and conflict and emotional break for their families (5–8). Therefore, finding ways to alleviate the infertility status and enhance fertility is desperate in those patients.

Hysterosalpingography (HSG) is first developed to screen and diagnose infertility (9). Interestingly, with the wide application of HSG in infertility patients, its fertility-enhancing effect is gradually recognized (1, 10, 11). However, this fertility-enhancing effect varies depending on the contrast agents used in the HSG procedure (10–12). In recent literature, the oil-based contrast agents exhibit superiority in increasing fertility in infertility patients over water-based contrast agents through balancing T helper (Th)1 immunity, altering membrane electronegativity, micro-viscosity, flushing effect, etc. (13–15). For instance, a multi-center randomized controlled study shows that using oil-based contrast agents during HSG increases the 10% natural pregnancy rate when compared with water-based contrast agents in a 6-month follow-up (1). Another study indicates that the oil-based agents might increase 5% of the ongoing pregnancy rate and 7.5% of the live birth rate meanwhile shortening the time to an ongoing pregnancy (10.0 vs. 13.7 months) in a 5-year follow-up duration (11). Even though the opinion about the fertility-enhancing effect of oil-based contrast agents is widely accepted, the variation of this effect with time is not fully understood.

Hence, this study aimed to evaluate fertility improvement by oil-based vs. water-based contrast agents during the HSG procedure and the change of this improvement during a 3-year follow-up.

## Materials and methods

### Patients

This study enrolled 1,000 female infertile patients who underwent HSG with oil- (*N* = 500) or water-based (*N* = 500) contrast agents in Nanjing Maternal and Child Health Hospital between January 2018 and June 2018. The patients who met the following criteria were eligible for the study: (i) aged 18–39 years old; (ii) met the indications for HSG; (iii) had regular ovulation; and (iv) continually tried to conceive without any forms of contraception during the follow-up period. The exclusion criteria were set as (i) previously received HSG examination before the study; (ii) patients who had absolute or relative contraindications to HSG; (iii) patients who had clearly non-tubal infertility, such as ovarian-related infertility (endocrine abnormalities and ovulation disorders), uterine-related infertility (serious abnormal uterine development, endometrial damage, intrauterine adhesion, endometrial polyps, submucosal fibroids, etc.); (iv) infertile for male causes; (v) patients who received infertility treatment during the follow-up period; (vi) patients who had complete obstruction or effusion of both fallopian tubes; and (vii) patients who were assessed as poor patency of fallopian tube based on HSG images (at least one fallopian tube was considered unobstructed but not smooth or unobstructed but not very smooth) according to Chinese Expert Consensus on Interventional Treatment of Fallopian Tube (2019). The patients who underwent HSG with the oil-based contrast agents were considered an oil-based group and those who experienced HSG with water-based contrast agents were considered a water-based group. The study was approved by the Ethics Committee of Nanjing Maternal and Child Health Hospital.

### Data documents

The clinical features, including age, times of pregnancy, times of delivery, duration of infertility, history of pelvic inflammation, history of endometriosis, history of tubal pregnancy, history of cesarean delivery, and history of other pelvic surgery, of all patients were recorded. In addition, the patients were followed up by telephone at 1 month (M1), 2 months (M2), 3 months (M3), 6 months (M6), 12 months (M12), 24 months (M24), and 36 months (M36) after HSG, and the pregnancy of patients was recorded for the further analysis.

### Hysterosalpingography procedures

**FIGURE 1:**
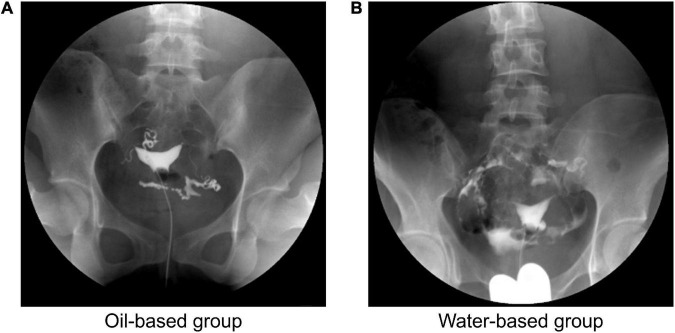
Image of hysterosalpingography (HSG) for typical cases. The typical image of injecting the oil-based contrast agents **(A)** or the water-based contrast agents **(B)** into the uterine cavity.

All eligible patients received an HSG examination performed 3–7 days after complete menstruation cessation using a digital gastrointestinal machine (FLEXAVISION, Shimadzu Corporation, Kyoto, Japan), and the procedure was the same as described in a previous study (16). In brief, after routine disinfection, the double-lumen balloon catheter was slowly inserted into the cervix and inflated with 2–3 ml of gases. Then, the oil-based contrast agents (ethiodized poppy seed oil, Jiangsu Hengrui Medicine Co., Ltd., Jiangsu, China) or the water-based contrast agents (ioversol injection, Jiangsu Hengrui Medicine Co., Ltd., Jiangsu, China) were slowly instilled into the uterine cavity (Figures 1A,B). The injection was continued until the uterine cavity was filled or significant discomfort occurred. During the infusion, the images of uterine cavity filling, fallopian tube filling, and pelvic spill were taken; the balloon was emptied, and the catheter was removed. Finally, the films of HSG were taken: for the oil-based group, delayed films were taken about 24 h later and for the water-based group, delayed films were taken about 15 min later. Based on the HSG images, the patency of the fallopian tube was assessed. After HSG, the patients were required to abstain from sex for 1 month due to radiation, intrauterine operation, and perioperative medication and then tried to conceive without contraception. Once the adverse event occurred, the HSG would stop, and patients might be asked to take a rest. If the symptom was not relieved, then a corresponding measure would be taken. For instance, if active vaginal bleeding occurred, the vagina tamponade would be carried out to facilitate the hemostasis and the anesthetic drug would be administrated.

### Assessment

The study assessed spontaneous pregnancy rate and duration between HSG and pregnancy. Spontaneous pregnancy was defined as a clinical intrauterine pregnancy (had positive fetal heartbeat confirmed by ultrasonographic examination) without any intervention after HSG (17); duration between HSG and spontaneous pregnancy was considered as the time interval from 1 month after HSG to positive urine pregnancy test (clinical intrauterine pregnancy was subsequently confirmed by ultrasonographic examination). Obvious abnormal pain was defined as a pain Visual Analogue Scale (VAS) score of more than 5.

### Statistics

SPSS V.20.0 software (IBM Corp., Armonk, NY, United States) was applied for statistical analysis, and GraphPad Prism V.7.02 software (GraphPad Software Inc., San Diego, CA, United States) was applied for graph construction. Metrological indexes were described using mean ± standard deviation (SD) or median [interquartile range (IQR)] as appropriate, and enumeration indexes were described using numbers with percentages [No. (%)]. The comparison of clinical features between two groups was analyzed using the Student’s *t*-test, the Chi-square test, Fisher’s exact test, or Wilcoxon rank-sum test. The cumulative pregnancy rate was elucidated using the Kaplan–Meier curve and determined using the log-rank test. Fertility-enhancing effect of HSG between groups was compared using odds ratio (OR) and 95% confidence interval (CI). OR was applied to compare the fertility-enhancing effect of HSG between groups. An OR value higher than 1 indicated that the fertility-enhancing effect of HSG was better in the oil-based group than the water-based group, and a higher OR value indicated the better fertility-enhancing effect of HSG. The multivariate Cox proportional hazards regression analysis was performed to evaluate the independent factors in predicting spontaneous pregnancy. Statistical significance was concluded if a two-sided *p*-value was less than 0.05.

## Results

### Clinical features

The median (IQR) age was 29.0 (24.3–32.0) years in the oil-based group and 27.0 (24.0–32.0) years in the water-based group (*p* = 0.195). Their infertile duration ranged from 1 to 7 years. None of the infertile patients in this study smoked currently; only 17 (3.4%) patients in the oil-based group and 14 (2.8%) patients in the water-based group had a smoke history (*p* = 0.584). In terms of body mass index (BMI), there was also no difference between these two groups (*p* = 0.977). Median (IQR) times of pregnancy and times of delivery were 0.9 (0.1–2.0) and 0.6 (0.2–1.3) in the oil-based group, and 0.6 (0.1–1.9) and 0.5 (0.1–1.3) in the water-based group (both *p* > 0.050). The median (IQR) duration of infertility was 2.0 (1.0–3.0) years in the oil-based group and 2.0 (1.0–3.0) years in the water-based group (*p* = 0.903). In addition, other clinical features [such as the history of pelvic inflammation (*p* = 0.388), history of endometriosis (*p* = 0.446), history of a tubal pregnancy (*p* = 0.302), history of cesarean delivery (*p* = 0.250), and history of other pelvic surgery (*p* = 0.660)], were of no difference between these two groups (Table 1).

**TABLE 1:** Clinical features.

Items	Oil-based group (*N* = 500)	Water-based group (*N* = 500)	*t/X ^2^/Z* value	*P*-value
Age (years), median (IQR)	29.0 (25.0–32.0)	27.0 (24.0–32.0)	–1.203	0.229
Current smoke	0 (0.0)	0 (0.0)	–	–
Smoke history	17 (3.4)	14 (2.8)	0.300	0.584
BMI			–0.029	0.977
BMI < 24	329 (65.8)	330 (66.0)		
24 ≤ BMI < 28	132 (26.4)	127 (25.4)		
BMI ≥ 28	39 (7.8)	43 (8.6)		
Times of pregnancy, median (IQR)	1.0 (0.0–2.0)	1.0 (0.0–2.0)	–1.331	0.183
Times of delivery, median (IQR)	0.0 (0.0–1.0)	0.0 (0.0–1.0)	–1.801	0.072
Duration of infertility (years), median (IQR)	2.0 (1.0–3.0)	2.0 (1.0–3.0)	–0.122	0.903
History of pelvic inflammation, No. (%)	88 (17.6)	82 (16.4)	0.255	0.613
History of endometriosis, No. (%)	52 (10.4)	49 (9.8)	0.099	0.753
History of tubal pregnancy, No. (%)	37 (7.4)	46 (9.2)	1.064	0.302
History of cesarean delivery, No. (%)	56 (11.2)	68 (13.6)	1.326	0.250
History of other pelvic surgery, No. (%)	26 (5.2)	23 (4.8)	0.084	0.772

IQR, interquartile range.

### Cumulative spontaneous pregnancy rate

**FIGURE 2:**
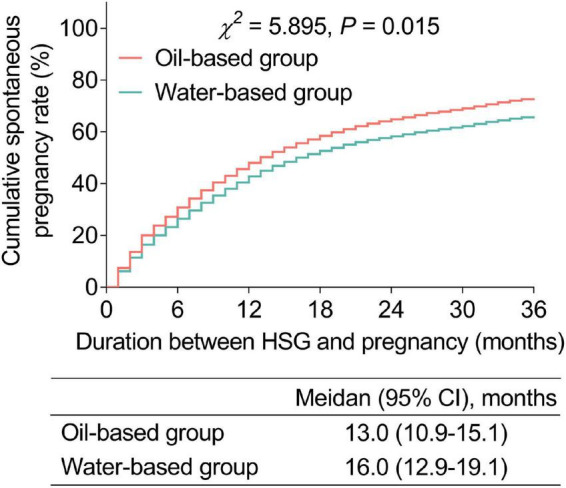
Cumulative spontaneous pregnancy rate. Comparison of the cumulative spontaneous pregnancy rate between the oil- and water-based groups.

The spontaneous pregnancy rate was 79.0% in the oil-based group and 70.2% in the water-based group within a 3-year follow-up after HSG. The median duration between HSG and pregnancy was 15.0 months (95% CI: 13.3–16.7 months) in the whole study subjects, 13.0 months (95% CI: 10.9–15.1 months) in the oil-based group, and 16.0 months (95% CI: 12.9–19.1 months) in the water-based group. By comparison with the log-rank test, the cumulative spontaneous pregnancy rate was increased in the oil-based group when compared with the water-based group (*p* = 0.015, Figure 2).

### Odds ratio for the improvement of cumulative spontaneous pregnancy rate in oil-based vs. water-based contrast agents

It was observed that the fertility-enhancing effect of HSG was better in the oil-based group when compared with the water-based group at 1 month (OR: 1.536; 95% CI: 0.970–2.432), 2 months (OR: 1.455; 95% CI: 1.056–2.003), 3 months (OR: 1.494; 95% CI: 1.151–1.938), 6 months (OR: 1.356; 95% CI: 1.124–1.636), 9 months (OR: 1.288; 95% CI: 1.106–1.500), 12 months (OR: 1.249; 95% CI: 1.098–1.420), 24 months (OR: 1.131; 95% CI: 1.036–1.234), and 36 months (OR: 1.125; 95% CI: 1.046–1.210). While this superiority of the fertility-enhancing effect of HSG in the oil-based group (compared with the water-based group) was decreased with the time frame (Table 2). Subgroup analysis showed that the patients without a history of endometriosis benefited more from the oil-based group at 6 months (OR: 1.878), 9 months (OR: 1.838), 12 months (OR: 1.771), 24 months (OR: 1.682), and 36 months (OR: 1.644; Supplementary Table 1).

**TABLE 2:** Cumulative spontaneous pregnancy rate over months.

Duration between HSG and pregnancy	Oil-based group No. (%)	Water-based group No. (%)	OR (95% CI)
One month	43 (8.6)	28 (5.6)	1.536 (0.970–2.432)
Two months	80 (16.0)	55 (11.0)	1.455 (1.056–2.003)
Three months	115 (23.0)	77 (15.4)	1.494 (1.151–1.938)
Six months	179 (35.8)	132 (26.4)	1.356 (1.124–1.636)
Nine months	228 (45.6)	177 (35.4)	1.288 (1.106–1.500)
Twelve months	271 (54.2)	217 (43.4)	1.249 (1.098–1.420)
Twenty-four months	355 (71.0)	314 (62.8)	1.131 (1.036–1.234)
Thirty-six months	395 (79.0)	351 (70.2)	1.125 (1.046–1.210)

HSG, hysterosalpingography; OR, odds ratio; CI, confidence interval.

The OR was re-analyzed based on the physiological cycles to minimize the difference in physiological cycles among all subjects. It also showed similar findings that the fertility-enhancing effect of HSG was better in the oil-based group when compared with the water-based group at one physiological cycle (OR: 1.481; 95% CI: 0.909–2.414), two physiological cycles (OR: 1.415; 95% CI: 0.995–2.012), three physiological cycles (OR: 1.372; 95% CI: 1.025–1.837), four physiological cycles (OR: 1.312; 95% CI: 1.006–1.712), five physiological cycles (OR: 1.277; 95% CI: 0.997–1.635), and six physiological cycles (OR: 1.258; 95% CI: 0.997–1.586). While this superiority of the fertility-enhancing effect of HSG in the oil-based group (compared with the water-based group) also showed a decreasing trend with the time frame (Table 3). After adjusting the potential confounders by multivariate Cox’s proportional hazards regression analysis, it was shown that the water-based group (vs. oil-based group) was independently associated with a declined spontaneous pregnancy rate (*p* = 0.004) (Supplementary Table 2).

**TABLE 3:** Cumulative spontaneous pregnancy rate over physiological cycles.

Duration between HSG and pregnancy	Oil-based group No. (%)	Water-based group No. (%)	OR (95% CI)
One physiological cycle	40 (8.0)	27 (5.4)	1.481 (0.909–2.414)
Two physiological cycles	75 (15.0)	53 (10.6)	1.415 (0.995–2.012)
Three physiological cycles	107 (21.4)	78 (15.6)	1.372 (1.025–1.837)
Four physiological cycles	126 (25.2)	96 (19.2)	1.312 (1.006–1.712)
Five physiological cycles	143 (28.6)	112 (22.4)	1.277 (0.997–1.635)
Six physiological cycles	161 (32.2)	128 (25.6)	1.258 (0.997–1.586)

HSG, hysterosalpingography; OR, odds ratio; CI, confidence interval.

### Adverse events

In total, 33 (6.6%) patients in the oil-based group and 36 (7.2%) patients in the water-based group showed obvious abnormal pain (*p* = 0.446). In addition, 3 (0.6%) patients in the oil-based group and 5 (1.0%) patients in the water-based group revealed active vaginal bleeding (*p* = 0.302; Supplementary Table 3).

## Discussion

In this study, we found that (1) the cumulative spontaneous pregnancy rate was increased in the oil-based group when compared with the water-based group; and (2) the superiority of the fertility-enhancing effect of HSG in the oil-based group (compared with the water-based group) showed a decreasing trend with the time frame.

The fertility-enhancing effect of HSG using oil-based contrast agents has been noticed in several studies. For instance, the water vs. oil (H2Oil trial) shows that the ongoing pregnancy rate (39.7% vs. 29.1%) and live births rate (38.8% vs. 28.1%) are increased in the oil-based group when compared with the water-based group (1). Meanwhile, two meta-analyses also verify this finding (10, 12). In line with previous studies, in this study, we found that the cumulative spontaneous pregnancy rate was increased in the oil-based group when compared with the water-based group, which could be explained as follows: (1) the HSG procedure with oil-based contrast agents indicated a tubal-flushing effect, which might be effective for removing the mucous plug and the fragment of impaired tubal tissue, therefore, increasing the fertility in infertile women (18); (2) oil-based contrast agents might regulate the immune environment (such as regulating the Th1/Th2 cell proportion; decreasing macrophage phagocytosis and adherence; and decreasing amount of dendritic cells), subsequently, reduced sperm phagocytosis and increased the fertility rate (13–15).

Even though it is shown that the oil-based contrast agents disclose an improvement of a fertility-enhancing effect than the water-based contrast agents, it is seldom reported how this improvement changes during a long-term follow-up period. Currently, we only retrieved the study conducted by van Welie et al. (19) on the time-dependent effect of oil-based contrast agents on reproductive outcomes in a non-Chinese female population. Our study showed that during a 3-year follow-up period, the oilbased group all showed a gain of a fertility-enhancing effect than the water-based group. However, this improvement was decreased with the time frame. Even though a re-analysis of this finding was based on the menstrual cycles, a similar result was also shown. These phenomena could be explained as follows: (1) many reasons might cause infertile in women, such as BMI, smoke (in men), maternal immune disorder, asthenozoospermia (from the male couple), and fallopian tube diseases (20–23). A single-time HSG with oil-based contrast agents might only improve some of these issues, including the maternal immune environment and flushing the tubules; apart from that, patients might also put forward some arrangements to cope with other infertile-related issues after HSG, such as losing weight, stopping smoking, and semen quality improvement. Therefore, in a subsequent post-treatment period, these arrangements might continuously improve the fertility of infertile women in both the oil-based group and water-based group, which means that the weight of the fertility-enhancing effect of these arrangements might increase, while the importance of the fertility-enhancing effect of oil-based contrast agents during HSG had decreased with the time frame. (2) Other post-HSG fertility-enhancing treatments (such as ovulation stimulation and traditional Chinese medicine) might also affect the spontaneous pregnancy rate. Therefore, the influence of spontaneous pregnancy rate by oil-based contrast agents was attenuated (24–27).

The novelty of this study included two aspects: one is the fertility-enhancing effect of oil-based contrast when compared with water-based contrast, and another one is that the duration of this fertility-enhancing effect might decrease with the follow-up period. One issue should be clarified in this study because the diagnostic laparoscopy was an invasive surgery, the diagnostic laparoscopy was not recommended to perform on infertile patients for the routine screening of etiology of infertile in China; therefore, the diagnostic laparoscopy was not performed on each patient in this study. The method for excluding non-tubal causes of infertility in this study was only if the patients with clearly non-tubal infertility (including ovarian-related infertility, endocrine abnormalities, and ovulation disorders), and uterine-related infertility (serious abnormal uterine development, endometrial damage, intrauterine adhesion, endometrial polyps, submucosal fibroids, etc.), they were excluded from this study. Another aspect should also be mentioned in the present study, i.e., ethiodized poppy seed oil used in the present study disclosed an intermediate viscosity between the traditional oil contrast medium and water-based contrast medium, which allowed it to disclose an intermediate diffusion speed from the uterine cavity to the pelvic cavity. There are some limitations to this study, including (1) the sample size was relatively small in the present study; thus, these findings should be verified in the bigger-sample-size survey; (2) the included infertile patients were relatively young, and their disease etiology was not complicated. Therefore, the study finding might be unsuitable for infertile patients with complicated etiology. (3) Only the spontaneous pregnancy rate was recorded in this study, while the live birth rate was hard to obtain; therefore, further research focusing on the live birth rate is needed.

Another issue should be mentioned, i.e., even though this study did not set the inclusion or exclusion criteria to exclude the patients with pituitary diseases, these patients were indeed excluded from this study. Because, patients with pituitary diseases were always complicated with diseases (such as hyperprolactinemia, menstrual disorders, endocrine abnormalities, and ovulation disorders) that might affect normal ovulation. However, according to the inclusion criteria (iii) “patients who had regular ovulation,” these patients could not be enrolled in this study. Therefore, these patients with pituitary diseases indeed were excluded.

In conclusion, the HSG procedure with oil-based contrast agents shows a fertility-enhancing effect when compared with the water-based contrast agents, and this improvement could last for at least 1 year. This finding indicates that the nature of pregnancy after the oil-based HSG could be achieved in those infertile couples complicated with mild tubal disease and without other etiology for infertile; therefore, the medical intervention for those infertile patients should be delayed after 1–2 years of preparing spontaneous pregnancy. The oil-based HSG procedure could be regarded as not only an examination method but also a therapy method for infertile, which should be recommended in infertile patients with HSG indication.

## Data availability statement

The original contributions presented in this study are included in the article/Supplementary material, further inquiries can be directed to the corresponding authors.

## Ethics statement

The studies involving human participants were reviewed and approved by the Ethics Committee of Nanjing Maternal and Child Health Hospital. The patients/participants provided their written informed consent to participate in this study.

## Authors contributions

JL contributed to the conception and design of the study, project development, data collection, and manuscript writing. DQ was responsible for data collection. WX contributed to project development. All authors have read and approved the final manuscript.
