# Corrigendum: Fertility-enhancing effect of oil-based contrast agents during hysterosalpingography and the variation of this effect within a 3-year follow-up period in infertile patients

**DOI:** 10.3389/fmed.2023.1325057

**Published:** 2023-11-16

**Authors:** Jingyuan Lu, Dan Qi, Wenjian Xu

**Affiliations:** ^1^Department of Radiological Intervention, Nanjing Maternity and Child Health Care Hospital, Women's Hospital of Nanjing Medical University, Nanjing, China; ^2^Department of Traditional Chinese Medicine, Nanjing Maternity and Child Health Care Hospital, Women's Hospital of Nanjing Medical University, Nanjing, China

**Keywords:** hysterosalpingography, oil-based contrast agents, water-based contrast agents, spontaneous pregnancy rate, infertile patients

In the published article, there was some errors in Table 1 as published. Upon reviewing the original data, it was found that a small portion of the data in age, times of pregnancy, times of delivery, history of pelvic inflammation, history of endometriosis, and history of other pelvic surgery contained inaccuracies. The updated table now reflects the accurate data, and we have recalculated the relevant statistics accordingly.

The corrected Table 1 and its caption appear below.

**Table 1:** Clinical features.

**Items**	**Oil-based group (*N* = 500)**	**Water-based group (*N* = 500)**	***t*/*X*^2^/*Z* value**	***P*-value**
Age (years), median (IQR)	29.0 (25.0–32.0)	27.0 (24.0–32.0)	−1.203	0.229
Current smoke	0 (0.0)	0 (0.0)	–	–
Smoke history	17 (3.4)	14 (2.8)	0.300	0.584
BMI			−0.029	0.977
BMI < 24	329 (65.8)	330 (66.0)		
24 ≤ BMI < 28	132 (26.4)	127 (25.4)		
BMI ≥ 28	39 (7.8)	43 (8.6)		
Times of pregnancy, median (IQR)	1.0 (0.0–2.0)	1.0 (0.0–2.0)	−1.331	0.183
Times of delivery, median (IQR)	0.0(0.0–1.0)	0.0(0.0–1.0)	−1.801	0.072
Duration of infertility (years), median (IQR)	2.0 (1.0–3.0)	2.0 (1.0–3.0)	−0.122	0.903
History of pelvic inflammation, No. (%)	88 (17.6)	82(16.4)	0.255	0.613
History of endometriosis, No. (%)	52 (10.4)	49 (9.8)	0.099	0.753
History of tubal pregnancy, No. (%)	37 (7.4)	46 (9.2)	1.064	0.302
History of cesarean delivery, No. (%)	56 (11.2)	68 (13.6)	1.326	0.250
History of other pelvic surgery, No. (%)	26 (5.2)	24 (4.8)	0.084	0.772

IQR, interquartile range.

In the published article, we missed to include a reference. The article “How long does the fertility-enhancing effect of hysterosalpingography with oil-based contrast last?” authored by van Welie et al. (19) addresses a subject that closely aligns with our research. Their study offers insights that resonate with our own investigation. We have added the reference, and cited it in the discussion section.

The sentence “Currently, we only retrieved the study conducted by van Welie et al. on the time-dependent effect of oil-based contrast agents on reproductive outcomes in a non-Chinese female population (19).” has been added to the **Discussion**, paragraph 3.

The corrected paragraph appears below:

“Even though it is shown that the oil-based contrast agents disclose an improvement of a fertility-enhancing effect than the water-based contrast agents, it is seldom reported how this improvement changes during a long-term follow-up period. Currently, we only retrieved the study conducted by van Welie et al. (19) on the time-dependent effect of oil-based contrast agents on reproductive outcomes in a non-Chinese female population. Our study showed that during a 3-year follow-up period, the oil-based group all showed a gain of a fertility-enhancing effect than the water-based group. However, this improvement was decreased with the time frame. Even though a re-analysis of this finding was based on the menstrual cycles, a similar result was also shown. These phenomena could be explained as follows: (1) many reasons might cause infertile in women, such as BMI, smoke (in men), maternal immune disorder, asthenozoospermia (from the male couple), and fallopian tube diseases (20–23). A single-time HSG with oil-based contrast agents might only improve some of these issues, including the maternal immune environment and flushing the tubules; apart from that, patients might also put forward some arrangements to cope with other infertile-related issues after HSG, such as losing weight, stopping smoking, and semen quality improvement. Therefore, in a subsequent post-treatment period, these arrangements might continuously improve the fertility of infertile women in both the oil-based group and water-based group, which means that the weight of the fertility-enhancing effect of these arrangements might increase, while the importance of the fertility-enhancing effect of oil-based contrast agents during HSG had decreased with the time frame. (2) Other post-HSG fertility-enhancing treatments (such as ovulation stimulation and traditional Chinese medicine) might also affect the spontaneous pregnancy rate. Therefore, the influence of spontaneous pregnancy rate by oil-based contrast agents was attenuated (24–27).”

The authors apologize for these errors and state that this does not change the scientific conclusions of the article in any way. The original article has been updated.
