# Supplementary material for: Fertility-enhancing effect of oil-based contrast agents during hysterosalpingography and the variation of this effect within a 3-year follow-up period in infertile patients
Source: Front Med (Lausanne). 2022 Aug 29;9:948945. doi: 10.3389/fmed.2022.948945 (PMC9473147; doi:10.3389/fmed.2022.948945)
Supplement: Supplementary file 1 [file Table_1.pdf]

Supplementary Materials

Supplementary Table 1. Cumulative spontaneous pregnancy rate over months in oil-based group.

Duration between HSG and pregnancy	Non- endometriosis (n = 450) No. (%)	Endometriosis (n = 50) No. (%)	OR (95% CI)
One month	41 (9.1)	2 (4.0)	2.278 (0.568-9.135)
Two months	76 (16.9)	4 (8.0)	2.111 (0.807-5.525)
Three months	109 (24.2)	6 (12.0)	2.019 (0.936-4.352)
Six months	169 (37.6)	10 (20.0)	1.878 (1.065-3.311)
Nine months	215 (47.8)	13 (26.0)	1.838 (1.140-2.962)
Twelve months	255 (56.7)	16 (32.0)	1.771 (1.173-2.674)
Twenty-four months	333 (74.0)	22 (44.0)	1.682 (1.224-2.310)
Thirty-six months	370 (82.2)	25 (50.0)	1.644 (1.242-2.177)

HSG, hysterosalpingography; OR, odds ratio; CI, confidence interval.

Supplementary Table 2. Multivariate Cox's proportional hazards regression analysis for spontaneous pregnancy.

Items	*P* value	HR	95%CI	
			Lower	Upper
Group (water-based vs. oil-based)	0.004	0.806	0.695	0.935
Age	0.718	1.003	0.988	1.017
Longer duration of infertility	<0.001	0.840	0.782	0.902
Times of pregnancy	0.282	0.827	0.584	1.170
Times of delivery	0.188	1.471	0.828	2.615
History of endometriosis	0.004	0.670	0.510	0.879
History of pelvic inflammation	0.114	0.850	0.695	1.040
History of tubal pregnancy	0.600	0.927	0.698	1.231
History of cesarean delivery	0.698	0.956	0.759	1.203
History of other pelvic surgery	0.677	1.070	0.777	1.473

HR, hazard ratio; CI, confidence interval.

Supplementary Table 3. Adverse event during the HSG.

Adverse events	Oil-based group (N = 500)	Water-based group (N = 500)	X^2 value	*P-value*
Obvious abnormal pain, No. (%)	33 (6.6)	36 (7.2)	0.581	0.446
Active vaginal bleeding, No. (%)	3 (0.6)	5 (1.0)	1.064	0.302

HSG, hysterosalpingography.
